# Large-scale prediction of long disordered regions in proteins using random forests

**DOI:** 10.1186/1471-2105-10-8

**Published:** 2009-01-07

**Authors:** Pengfei Han, Xiuzhen Zhang, Raymond S Norton, Zhi-Ping Feng

**Affiliations:** 1 School of Computer Science and IT, RMIT University, Melbourne, Victoria 3001, Australia; 2 The Walter and Eliza Hall Institute of Medical Research, Parkville, Victoria 3052, Australia

## Abstract

**Background:**

Many proteins contain disordered regions that lack fixed three-dimensional (3D) structure under physiological conditions but have important biological functions. Prediction of disordered regions in protein sequences is important for understanding protein function and in high-throughput determination of protein structures. Machine learning techniques, including neural networks and support vector machines have been widely used in such predictions. Predictors designed for long disordered regions are usually less successful in predicting short disordered regions. Combining prediction of short and long disordered regions will dramatically increase the complexity of the prediction algorithm and make the predictor unsuitable for large-scale applications. Efficient batch prediction of long disordered regions alone is of greater interest in large-scale proteome studies.

**Results:**

A new algorithm, IUPforest-L, for predicting long disordered regions using the random forest learning model is proposed in this paper. IUPforest-L is based on the Moreau-Broto auto-correlation function of amino acid indices (AAIs) and other physicochemical features of the primary sequences. In 10-fold cross validation tests, IUPforest-L can achieve an area of 89.5% under the receiver operating characteristic (ROC) curve. Compared with existing disorder predictors, IUPforest-L has high prediction accuracy and is efficient for predicting long disordered regions in large-scale proteomes.

**Conclusion:**

The random forest model based on the auto-correlation functions of the AAIs within a protein fragment and other physicochemical features could effectively detect long disordered regions in proteins. A new predictor, IUPforest-L, was developed to batch predict long disordered regions in proteins, and the server can be accessed from

## Background

Intrinsically unstructured/disordered proteins (IUPs/IDPs) contain long disordered regions or are completely disordered [1]. IUPs are abundant in higher organisms and often involved in key biological processes, such as transcriptional and translational regulation, membrane fusion and transport, cell-signal transduction, protein phosphorylation, the storage of small molecules and the regulation of self-assembly of large multi-protein complexes [2-11]. The disordered state in IUPs creates larger intermolecular interfaces [12], which increase the speed of interaction with potential binding partners even in the absence of tight binding, and provide flexibility for binding diverse ligands [2,5,11,13-15]. However, long disordered regions in IUPs cause difficulties in protein structure determination by both X-ray crystallography and nuclear magnetic resonance (NMR) spectroscopy. Efficient prediction of disordered region(s) in IUPs by computational methods can provide valuable information in high-throughput protein structure characterization, and reveal useful information on protein function [15].

Many predictors have been developed to predict disordered regions in proteins, such as PONDR [16], RONN [17,18], VL2, VL3, VL3H and VL3E from DisProt [1,19,20], NORSp [21,22], DISpro [23], FoldIndex [24], DISOPRED and DISOPRED2 [25-27], GlobPlot [28] and DisEMBL [29], IUPred [30], Prelink [31], DRIP-PRED (MacCallum, online publication ), FoldUnfold [32], Spritz [33], DisPSSMP [34], VSL1 and VSL2 [35,36], POODLE-L [37], POODLE-S [38], Ucon [39], PrDOS and metaPrDOS [40,41]. Among these predictors, neural networks and support vector machines (SVM) are widely used machine learning models.

The accuracy of disorder predictors is generally limited by the existence of various kinds of disorder which are represented unevenly in the various databases, and the lack of a unique definition of disorder [30]. Predictors designed for long disordered regions are usually less successful in predicting short disordered regions [36,42] because the long and short disordered regions have different sequence features. As a result, some predictors are specified for predicting long disordered regions, such as POODLE-L [37], while predictors targeting all types of disordered regions usually have to sacrifice time efficiency for exploiting heterogeneous sequence properties, especially the evolution information extracted from PSI-BLAST or protein secondary structure [25,27,33-36,38].

In this paper, a new algorithm, IUPforest-L, is proposed for predicting long disordered regions based on the random forest learning model [43] and simple parameters extracted from the amino acid sequences and amino acid indices (AAIs) [44]. 10-fold cross validation tests and blind tests demonstrate that IUPforest-L can achieve significantly higher accuracy than many existing algorithms in predicting long disordered regions. The high efficiency of IUPforest-L makes it a suitable tool for high-throughput comparative proteomics studies.

## Methods

### Training and test datasets

To train IUPforest-L, a subset (positive training set) of disordered regions was constructed based on DisProt [20] (version3.6), which includes 352 regions of 30 aa or more in length, and 47251 aa in total. The negative training set was extracted from PDBSelect25 [45] (Oct. 2004 version), from which 366 sequences (80,324 aa in total) of at least 80 aa were selected. Each of them has a high resolution crystal structure (< 2.0Å), free from missing backbone or side chain coordinates and free from non-standard amino acid residues.

To assess the prediction performance of IUPforest-L, three datasets were used for blind tests. The first dataset was based on the dataset constructed by Hirose *et al *(Hirose-ADS1) as a blind test dataset of POODLE-L [37]. Hirose-ADS1 contains 53 ordered regions of at least 40 aa (11431 aa in total) from the Protein Data Bank [46] and 63 disordered regions of at least 30 aa (8700 aa in total) from DisProt (version 3.0). The second test set (Han-ADS1) comprised of 53 ordered regions as in Hirose-ADS1 and 33 long disordered regions (5959 aa in total) from the latest DisProt (version 4.8), after removing disordered regions homologous to those in DisProt (version 3.6) using the CD-HIT algorithm with a threshold of 0.9 sequence identity [47]. The third test set (Peng-DB) was constructed based on the blind test dataset of VLS2 [35], where 56 long disordered regions of at least 30 aa (2841 aa in total) and 1965 ordered regions (318431 aa in total) were used in the assessment. For an objective blind test of IUPforest-L on Hirose-ADS1 (as reported in Table 1), disordered and ordered regions homologous to those in Hirose-ADS1 were removed from our training set based on the CD-HIT algorithm with a threshold of 0.9 sequence identity [47], resulting in 293 disordered regions and 364 ordered regions for training the predictor. Similarly for an objective blind test on Han-ADS1 (as reported in Table 2), ordered regions homologous to the 53 ordered regions in Hirose-ADS1 were also removed from the original training set for training the predictor. The final IUPforest-L was still trained by the whole training set. Han-ADS1 is listed in the Additional file 1 and is also available online at .

### The random forest model

**Figure 1:**
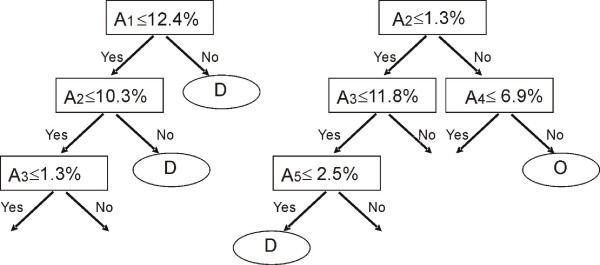
**A sample random forest**. In the decision tree on the left, the node at the root tests an attribute, such as the first order auto-correlation function of the normalized flexibility parameters (see below). If it is higher than a given threshold then the residue is in a disordered state (the right branch labelled D); otherwise another input attribute is tested and a set of other tests are further performed until a decision is made. A random forest can comprise hundreds of decision trees.

A random forest is an ensemble of unpruned decision trees (shown in Figure 1), where each tree is grown using a (bootstrap) subset of the training dataset [43]. Bootstrapping is a resampling technique where a number of bootstrap training sets are drawn randomly from the original training set with replacement. Each tree induced from bootstrap samples grows to full length and the number of trees in the forest is adjustable. To classify an instance of unknown class label, each tree casts a unit classification vote. The forest selects the classification having the most votes over all the trees in the forest. Compared with the decision tree classifier [48], random forests have better classification accuracy, are more tolerant to noise and are less dependent on the training datasets.

### Features used in training and test

When a window of *w *aa slides along a sequence, six types of features were derived from residues within the window, as defined and explained below.

1) Auto-correlation function of amino acid indices (AAIs)

Each residue in the training set was replaced with a value of the normalized amino acid index (AAI), which is a set of 20 numerical values representing the physicochemical and biological property of 20 amino acids chosen from the AAI Database [44]. As such, a sequence of *N *amino acids in the training set was firstly transformed into a numerical sequence [49,50], and denoted as:

(1) *P*_1_*P*_2 _⋯ *P*_*i *_⋯ *P*_*i*+*w *_⋯ *P*_*N*_

Then the sequences were smoothed with the Savitzky-Golay filter [51]. The Moreau-Broto auto-correlation function *F*_*d *_of an AAI was then calculated within a window, which is defined as:

(2) $$F_{d}=\frac{1}{w-d}\sum_{i=1}^{w-d}p_{i}\times p_{i+d},\;(d=1,2,...,w-1)$$

where *w *is the window size, *p*_*i *_and *p*_*i*+*d *_are the AAI values at positions *i *and *i+d *respectively [49,50]. For example, when *d *= 1, the numerical value for each residue (*i*) in the window multiplies by the value of the next nearby residue (*i+1*) and *F*_1 _is the average of these *w*-1 products. Similarly, *F*_2 _is the average of the *w*-2 products generated from every other residue. The value of *d *represented the order of the correlation and was tuned to optimize the prediction performance. The *F*_*d *_(*d *= 1, 2,..., 30) for the 40 sets of AAI listed in Table A1 in the Additional file 2 was calculated and evaluated in training IUPforest-L.

2) The mean hydrophobicity, defined as the average value of Kyte and Doolittle's hydrophobicity [52] in the window.

3) The modified hydrophobic cluster [31], calculated as the longest hydrophobic clusters in the window divided by the window size.

4) The mean net charge within the window and local mean net charge within a 13 aa fragment centered at the middle residue. Residues K and R were defined as +1; D and E were defined as -1; other residues were 0.

5) The mean contact number, defined as the mean expected number of contacts in the globular state of all residues within the window [53].

6) The composition of four reduced amino acid groups [48] and the Shannon's entropy (*K*_2_) of the amino acid composition within the window were calculated.

### IUPforest-L

**Figure 2:**
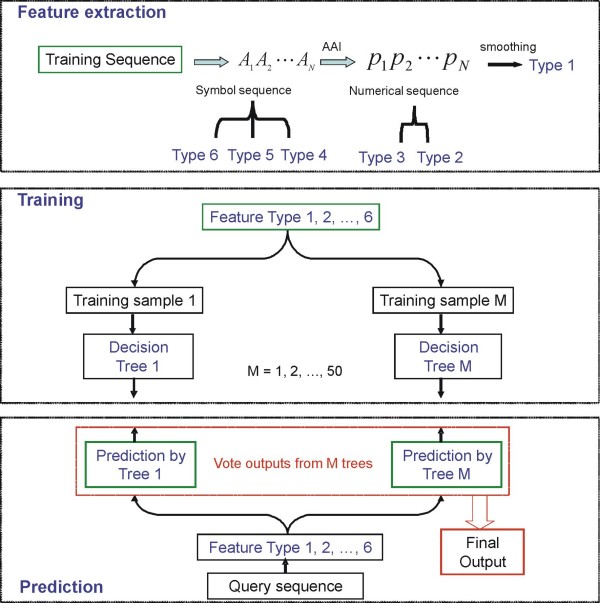
**Flow chart of IUPforest-L**. The sequence features were calculated when a window slides along a protein sequence. IUPforest-L models were trained from the six types of features and the prediction result could be obtained by each of the trees in the forest. The final score in the prediction was the combination of the outcomes from all trees by voting.

A flow chart of IUPforest-L is shown in Figure 2. At the training stage, features listed above were calculated when a window of *w *aa slides from the N-terminal end to the C-terminal end of a protein sequence. Each window was tagged with a label of disorder (Positive or P) or order (Negative or N) according to the label of the central residue, and IUPforest-L models were trained from the six types of features and the prediction result could be obtained by each of the trees in the forest. The final score was the combination of the outcomes from all trees by voting and smoothing [51]. A threshold that best classifies the ordered or disordered state of a residue could be defined based on the scores and the optimal evaluated values in the 10-fold cross validation tests.

During the prediction stage, the features were firstly calculated when a window slides over an inquiry sequence and then a probability score of a residue being disordered was assigned by IUPforest-L. A region was annotated as disordered only when 30 or more consecutive amino acid residues were predicted to be disordered.

### Evaluations

To estimate the generalization accuracy, 10-fold cross validation tests were conducted, where 90% of the sequences in the training set were randomly used in training and the other 10% were used in test. The process was repeated for the entire dataset and the final result was the average of the results from 10 processes. In addition, independent tests were performed on Hirose-ADS1 [38], Han-ADS1 and Peng-DB [35].

During the cross validation test, the confusion matrix, which comprises true positive (*TP*), false positive (*FP*), true negative (*TN*) and false negative (*FN*), was used to evaluate the prediction performance in terms of the following measures:

*1) AUC*, the area under the receiver operating characteristic (ROC) curve. Each point of a ROC curve was defined by a pair of values for the true positive rate ($\frac{TP}{TP+FN}$, or *sensitivit*y) and the false positive rate ($\frac{FP}{TN+FP}$, or *1-specificity*).

2) Balanced overall accuracy

(3) $$Bacc\equiv \frac{sensitivity+specificity}{2}$$

3) Sproduct

(4) *Sproduct *≡ *sensitivity × specificity*

4) Matthew's correlation functions (*MCC*)

(5) $$MCC\equiv \frac{TP\times TN-FP\times FN}{\sqrt{(TP+FP)\times (TP+FN)\times \left(TN+FP)\times (TN+FN)\right)}}$$

5) Sw

(6) $$Sw\equiv \frac{w_{disorder}\times TP-w_{order}\times FP+w_{order}\times TN-w_{disorder}\times FN}{w_{disorder}\times (TP+FN)+w_{order}\times (TN+FP)}$$

where *w*_*disorder *_and *w*_*order *_are the weights for disorder and order, respectively, that are inversely proportional to the number of residues in the disordered and ordered state. *Sw *is also referred to as *probability excess *[34].

The *Sproduct *and *Sw *scores were used in assessing the prediction of disordered residues in the Critical Assessment of techniques for protein Structure Prediction (CASP6 and CASP7) [54].

## Results

### 10-fold cross validation

**Figure 3:**
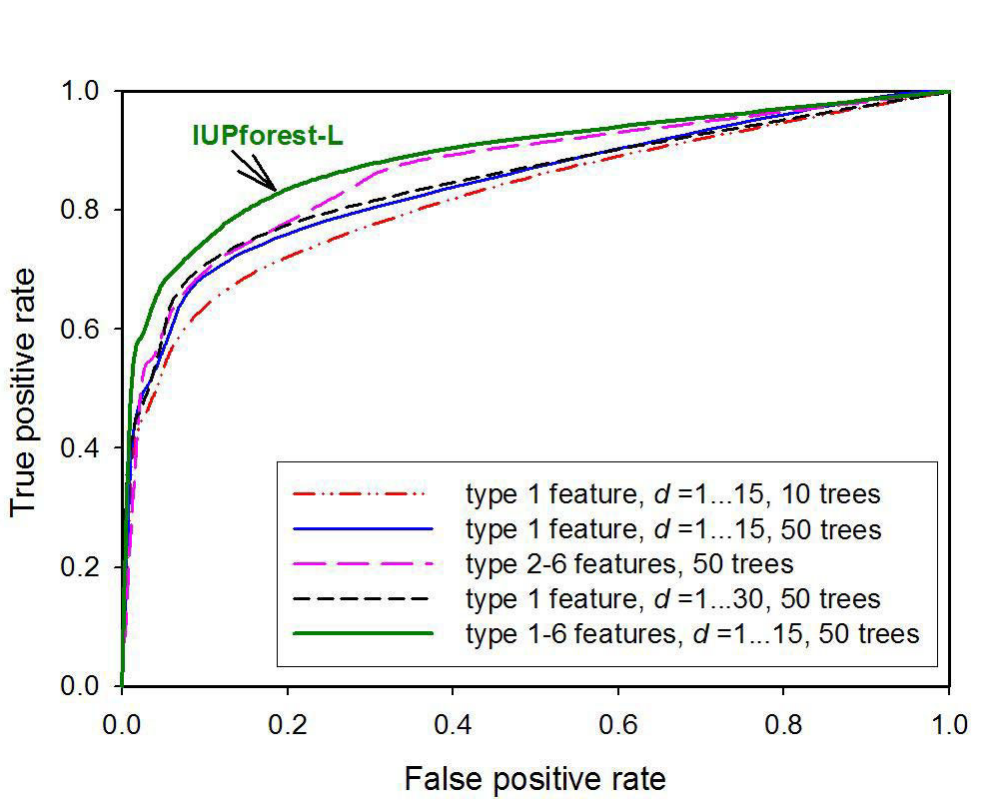
**ROC curves of 10-fold cross validation tests**. The ROC curves of IUPforest-L in 10-fold cross validation tests are shown. The IUPforest-L could reach a 76% true positive rate at a 10% false positive rate with *MCC *= 0.67, *Sproduct *= 0.64 and an area of 89.5% under the ROC curve on the training data set with a window of 31 aa.

The 10-fold cross validation test results using a window of 31 aa are shown in Figure 3. With the type 1 features (the auto-correlation function of AAIs), a forest of more trees has better predictive ability. For example, the *AUC *increased by 2% when the number of trees increased from 10 to 50. However, the prediction accuracy increased only modestly when the number of trees increased further from 50 to 100, while the training and prediction times increased significantly. Detailed test results on the time consumption with number of trees from 10 to 300 are shown in Additional file 3. The default setting of IUPforest-L is a forest of 50 trees for large-scale application.

With a forest of a fixed number of trees, the ROC curve trained with the auto-correlation function with *d *value between 1 and 15 almost overlaps with the ROC curve trained with *d *between 1 and 30. This result indicates that continuous correlations between nearby residues from 1 to 15 along the sequence could determine whether the fragment is involved in a long disordered region.

Figure 3 shows that training with either type 1 or the combination of type 2–6 features could reach the 70.5% or 70.0% true positive rates with a 10% false positive rate, while their combination of type 1–6 features could lead to a higher true positive rate of 76%, and an area of 89.5% under the ROC curve. This result indicates that type 1 and type 2–6 features have redundant, but complementary structural information. Type 2–6 features generated only nine parameters in total within a given window, while type 1 features could generate hundreds of parameters that take into account both order information and physicochemical properties. It has been shown that the random forest model has no risk of overfitting with an increasing number of trees when the input parameters increase [43]. As such, using type 1 features to train the random forest could extract more sequence-structure information [55] and it was thus conjectured that better prediction accuracy could be achieved with the auto-correlation functions generated from AAIs combined with other features of type 2–6.

The window size and step size for sliding the window are additional parameters for tuning the performance of IUPforest-L models. The window should be of a reasonable size so that the AAI-based correlation can be of significance within a reasonable training or test time. Training with small windows increases training time and can introduce noises, whereas training with large windows can lose local information. Our test results indicated that from window size of 19 aa to 47 aa, the random forest gave more stable result on blind test set Han-ADS1, but the accuracy on the 10-fold cross validation test on the training set will drop with larger window size (details listed in the Additional file 4). To batch predict long disordered regions, the window size of 31 aa was set in default to keep the balance between high efficiency and accuracy. The step size for sliding windows can also affect the accuracy and overall time efficiency at both the training and test stage. If the step size is too small, when a window slides along a sequence, it will introduce redundancy between windows and prolong the time for training models. Our experiments (details listed in the Additional file 4) show that with a sliding step of 20 aa (default setting) models achieve stable sensitivity without significantly prolonging the training process.

### Blind tests

**Figure 4:**
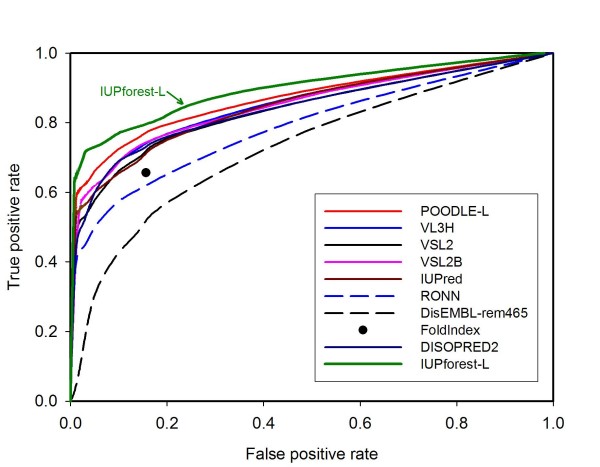
**ROC curves on test set Hirose-ADS1**. The ROC curves for IUPforest-L and nine publicly available predictors on the blind test dataset Hirose-ADS1 are shown. IUPforest-L has the best performance in terms of the *AUC*.

**Figure 5:**
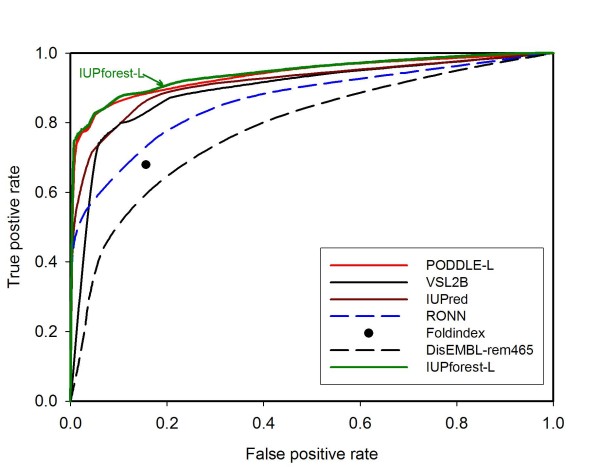
**ROC curves on test set Han-ADS1**. The ROC curves for IUPforest-L and some publicly available predictors on the blind test dataset Han-ADS1 are shown. IUPforest-L performs better in terms of the *AUC *than most of the predictors.

**Figure 6:**
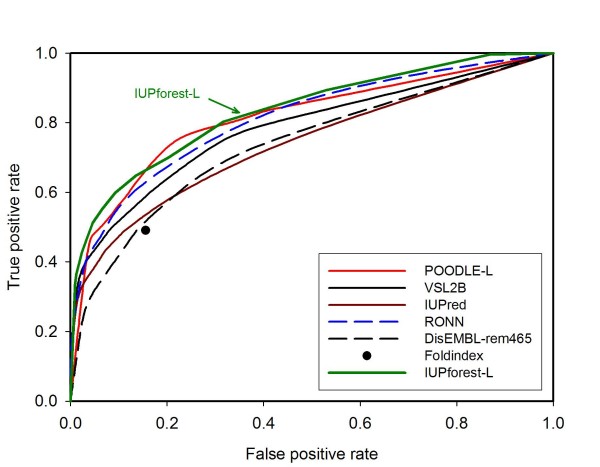
**ROC curves on test set Peng-DB**. The ROC curves for IUPforest-L and some publicly available predictors on the blind test dataset Peng-DB are shown. IUPforest-L performs better in terms of the *AUC *than most of the predictors.

Figure 4 depicts the ROC curves for IUPforest-L and nine other publicly available predictors on the blind test dataset Hirose-ADS1, including the most recently developed POODLE-L [37] and the well-established predictor VSL2 [35]. It is obvious that IUPforest-L outperforms most of the other predictors in terms of the *AUC *in predicting long disordered regions. At low false positive rates (< 10%), IUPforest-L achieves the highest sensitivity among all the predictors. In terms of other performance measures listed in Table 1, IUPforest-L is also comparable to or better than other predictors. Figure 5 and Table 2 show the result of comparisons of IUPforest-L with POODLE-L and other predictors on the Han-ADS1. It can be seen that IUPforest-L always performs better than most of them. Figure 6 and Table 3 shows the result of comparisons of IUPforest-L with POODLE-L and other predictors on the Peng-DB. It can be seen again that IUPforest-L always performs better than most of them.

**Table 1:** Comparison of IUPforest-L with other predictors on th test set Hirose-ADS1*

Measure(%)	*sen*	*spe*	*MCC*	*Spro*	*Bacc*	*Sw*	*AUC*
**IUPforest-L**	**72.0**	**93.4**	**72.3**	**68.7**	**84.3**	**68.7**	**90.0**
DisEMBL	24.5	96.4	31.2	23.6	60.5	20.9	73.3
DISOPRED2	63.5	93.9	61.6	59.6	78.7	57.4	84.8
FoldIndex	62.2	84.4	48.1	52.5	73.3	46.6	N/A
FoldUnfold	59.8	95.9	61.4	57.4	77.9	55.7	N/A
IUPred	59.5	95.6	60.7	56.9	77.6	55.1	85.3
POODLE-L	66.9	94.9	65.8	63.5	80.9	61.8	87.3
RONN	62.8	83.7	47.8	52.5	73.3	46.5	79.7
Spritz (long)	16.5	92.5	13.9	15.2	54.5	-2.65	N/A
VL3H	73.4	85.8	60.0	63.0	79.6	59.2	85.6
VSL2	75.5	79.4	54.7	59.9	77.5	54.9	84.4
VSL2B	60.9	83.0	60.9	59.1	79.6	59.1	85.4

+ Results for VL3H, VSL2, DISOPRED2, FoldUnfold were obtained from [37]. N/A means no raw score was available for comparison.

**Table 2:** Comparison of IUPforest-L with other predictors on test set Han-ADS1*

Measure(%)	*sen*	*spe*	*MCC*	*Spro*	*Bacc*	*Sw*	*AUC*
**IUPforest-L**	**87.5**	**94.4**	**82.3**	**82.6**	**91.0**	**82.1**	**94.3**
DisEMBL	43.9	93.2	44.4	40.9	68.5	37.8	76.5
DISOPRED2	63.1	96.2	65.8	60.7	79.7	60.2	90.0
FoldIndex	67.9	84.4	52.5	57.3	76.2	52.7	N/A
FoldUnfold	85.5	87.7	71.0	75.0	86.6	73.4	N/A
IUPred	71.4	95.6	71.4	68.3	83.5	67.6	92.0
POODLE-L	82.2	94.9	78.8	78.0	88.6	77.4	94.0
RONN	54.9	96.7	60.2	53.1	75.8	52.6	86.6
Spritz (long)	62.1	96.7	65.8	60.0	79.4	59.3	N/A
VL3H	66.4	96.1	68.3	63.8	81.3	63.2	94.5
VSL2	87.1	89.3	75.1	77.8	88.2	76.4	84.4
VSL2B	70.4	94.8	69.3	66.8	82.6	65.8	91.0

* N/A means no raw score was available for comparison.

**Table 3:** Comparison of IUPforest-L with other predictors on test set Peng-DB.*

Measure(%)	*sen*	*spe*	*MCC*	*Spro*	*Bacc*	*Sw*	*AUC*
**IUPforest-L**	**39.1**	**98.3**	**24.8**	**38.4**	**68.7**	**41.0**	**83.2**
DisEMBL	43.2	89.9	10.1	38.8	66.5	33.1	73.7
DISOPRED2	40.9	96.7	19.3	39.5	68.8	39.6	80.0
FoldIndex	49.1	84.4	85.8	41.4	66.7	35.7	N/A
FoldUnfold	37.9	90.9	31.0	34.5	64.4	19.0	N/A
IUPred	43.2	92.8	12.8	40.1	68.0	36.1	75.2
POODLE-L	39.9	96.8	18.6	38.9	68.3	36.7	79.8
RONN	42.3	96.3	18.3	40.7	69.3	38.6	79.2
Spritz (long)	23.1	96.9	18.6	22.4	60.0	-19	N/A
VL3H	40.2	96.8	11.8	38.9	68.5	65.6	91.3
VSL2	58.2	92.9	14.6	54.1	75.6	61.0	85.4
VSL2B	42.2	95.7	16.9	40.4	69.0	38.0	78.9

*A random sample of 112 sequences from the ordered test set of Peng-DB was used to test Spritz(long) and DISOPRED2 due to the size restriction of the prediction services. N/A means no raw score was available for comparison.

## Discussion

Protein structures are stabilized by numerous intramolecular interactions such as hydrophobic, electrostatic, van der Waals, and hydrogen bonds. The autocorrelation function tests whether the physicochemical property of one residue is independent of that of neighbouring residues. A group of residues involved in ordered structure close to other groups of residues in space will be dynamically constrained by the backbone or side chain interactions from these residues, and hence the residues in both groups will show higher density in the contact map or have higher pairwise correlation. On the other hand, a repetitive sequence of amino acids can also give significant positive correlation for all physicochemical properties. Therefore, residues within a fragment exhibiting a higher autocorrelation may either be structurally constrained, or have low sequence complexity. The random forest learning model employed by the IUPforest-L disorder predictor combines the complementary contributions from the autocorrelation function (type 1 feature) and other types of features, so that structural information is extracted with a high degree of prediction accuracy.

The random forest model is an ensemble learning model and is known to be more robust to noise than many non-ensemble learning models. However, as a classifier based on the random forest needs to load many decision trees into memory, it is relatively slow for a forest to predict a single instance at a time. As a result, the current web server of IUPforest-L is better suited to batch prediction of a large number of protein sequences, which provides an alternative useful tool in large-scale analysis of long disordered regions in proteomics. As an initial application, we have provided a server, IUPforest-L, for batch protein sequences analysis with the output of overall summary and details for each sequence. For convenience in proteomic comparisons, the prediction results for 62 eukaryotes linked to The European Bioinformatics Institute are also pre-calculated and can be downloaded from the server.

## Conclusion

IUP studies are important because disordered regions are common and functionally important in proteins. The new features, the auto-correlation functions of AAIs within a protein fragment, reflect both residues' contact information and sequence complexity. The random forest model based on this new type of features and other physicochemical features could effectively detect long disordered regions in proteins. As a result, a new predictor, IUPforest-L, was developed to predict long disordered regions in proteins. Its high accuracy and high efficiency make it a useful tool in large-scale protein sequence analysis.

## Authors' contributions

PH wrote up the computer program, carried out calculations and developed the web interface; RSN participated in design of the project and drafting the manuscript; XZ and ZPF participated in design of the project, development of the algorithm, interpretation of the results and drafting the manuscript.

## Supplementary Material

Additional file 1 **Blind test set Han-ADS1**. 53 ordered regions and 33 disordered regions longer than 30 aa used in blind test IUPforest-L. Click here for file

Additional file 2 **Table A1**: The amino acid indices (AAIs) used in the study. The names of 20 disorder-correlated indices and 20 ordered-correlated indices. Click here for file

Additional file 3 **Influence of the number of trees and time efficiency**. Result and discussion on the influence of the number of trees and time efficiency. Click here for file

Additional file 4 **Influence of the windows and sliding step for training IUPforest-L**. Results and discussions on the influence of the windows and sliding step for training IUPforest-L. Click here for file
