# Supplementary material for: Large-scale prediction of long disordered regions in proteins using random forests
Source: BMC Bioinformatics. 2009 Jan 7;10:8. doi: 10.1186/1471-2105-10-8 (PMC2637845; doi:10.1186/1471-2105-10-8)
Supplement: Additional file 2 — Table A1: The amino acid indices (AAIs) used in the study. The names of 20 disorder-correlated indices and 20 ordered-correlated indices. [file 1471-2105-10-8-S2.pdf]

Table A1. The amino acid indices (AAIs) used in this study.

(A) Disorder-correlated indices

VINM940101	Normalized flexibility parameters (B-values), average (Vihinen, Torkkila et al. 1994)
VINM940102	Normalized flexibility parameters (B-values) for each residue surrounded by none rigid neighbours (Vihinen, Torkkila et al. 1994)
VINM940103	Normalized flexibility parameters (B-values) for each residue surrounded by one rigid neighbours (Vihinen, Torkkila et al. 1994)
MIYS990101	Relative partition energies derived by the Bethe approximation (Miyazawa and Jernigan 1999)
MIYS990102	Optimized relative partition energies method A (Miyazawa and Jernigan 1999)
MIYS990103	Optimized relative partition energies - method B (Miyazawa and Jernigan 1999)
MIYS990104	Optimized relative partition energies - method C (Miyazawa and Jernigan 1999)
MIYS990105	Optimized relative partition energies - method D (Miyazawa and Jernigan 1999)
MEIH800101	Average reduced distance for C-alpha (Meirovitch 1980)
OOBM770103	Long range non-bonded energy per atom (Oobatake and Ooi 1977)
PARJ860101	HPLC parameter (Parker, Guo et al. 1986)
BULH740101	Transfer free energy to surface (Bull and Breese 1974)
MUNV940103	Free energy in beta-strand conformation (Munoz and Serrano 1994)
FASG890101	Hydrophobicity index (Fasman 1989)
PUNT030102	Knowledge-based membrane-propensity scale from 3D_Helix in MPtopo databases (Punta and Maritan 2003)
GUYH850102	Apparent partition energies calculated from Wertz-Scheraga index (Guy 1985)
CHOP780203	Normalized frequency of beta-turn (Chou and Fasman 1978)
WOLS870101	Principal property value z1 (Wold 1987)
PARS000101	p-Values of mesophilic proteins based on the distributions of B values (Parthasarathy and Murthy 2000)
RACS770101	Average reduced distance for C-alpha (Rackovsky and Scheraga 1977)

(B) Order-correlated indices

BASU050101	Interactivity scale obtained from the contact matrix (Bastolla, Porto et al. 2005)
BASU050102	Interactivity scale obtained by maximizing the mean of correlation coefficient over single-domain globular proteins (Bastolla, Porto et al. 2005)
BASU050103	Interactivity scale obtained by maximizing the mean of correlation coefficient over pairs of sequences sharing the TIM barrel fold (Bastolla, Porto et al. 2005)
CIDH920101	Normalized hydrophobicity scales for alpha-proteins (Cid, Bunster et al. 1992)
CIDH920102	Normalized hydrophobicity scales for beta-proteins (Cid, Bunster et al. 1992)
CIDH920103	Normalized hydrophobicity scales for alpha+beta-proteins (Cid, Bunster et al. 1992)
CIDH920104	Normalized hydrophobicity scales for alpha/beta-proteins (Cid, Bunster et al. 1992)
CIDH920105	Normalized average hydrophobicity scales (Cid, Bunster et al. 1992)
ZHOH040101	The stability scale from the knowledge-based atom-atom potential (Zhou and Zhou 2004)
ZHOH040102	The relative stability scale extracted from mutation experiments (Zhou and Zhou 2004)
ZHOH040103	Buriability (Zhou and Zhou 2004)
NOZY710101	Transfer energy, organic solvent/water (Nozaki and Tanford 1971)
PONP930101	Hydrophobicity scales (Ponnuswamy 1993)
MANP780101	Average surrounding hydrophobicity (Manavalan 1978)
VENT840101	Bitterness (Venanzi 1984)
NISK860101	14 A contact number (Nishikawa and Ooi 1986)
PTIO830102	Beta-coil equilibrium constant (Ptitsyn and Finkelstein 1983)
NADH010105	Hydropathy scale based on self-information values in the two-state mode (Naderi-Manesh, Sadeghi et al. 2001)
LIFS790101	Conformational preference for all beta-strands (Lifson and Sander 1979)
PONP800101	Surrounding hydrophobicity in folded form (Ponnuswamy, Prabhakaran et al. 1980)

Reference:

Bastolla, U., M. Porto, et al. (2005). "Principal eigenvector of contact matrices and hydrophobicity profiles in proteins." Proteins **58**(1): 22-30.

Bull, H. B. and K. Breese (1974). "Surface tension of amino acid solutions: a hydrophobicity scale of the amino acid residues." Arch Biochem Biophys **161**(2): 665-70.

Chou, P. Y. and G. D. Fasman (1978). "Prediction of the secondary structure of proteins from their amino acid sequence." Adv Enzymol Relat Areas Mol Biol **47**: 45-148.

Cid, H., M. Bunster, et al. (1992). "Hydrophobicity and structural classes in proteins." Protein Eng **5**(5): 373-5.

Fasman, G. D. (1989). "Prediction of Protein Structure and the Principles of Protein Conformation." Plenum, New York 1989, page 457, Table XVII.

Guy, H. R. (1985). "Amino acid side-chain partition energies and distribution of residues in soluble proteins." Biophys J **47**(1): 61-70.

Lifson, S. and C. Sander (1979). "Antiparallel and parallel beta-strands differ in amino acid residue preferences." Nature **282**(5734): 109-11.

Manavalan, P. a. P., P.K. (1978). "Hydrophobic character of amino acid residues in globular proteins." Nature **275**: 673-674.

Meirovitch, H., Rackovsky, S. and Scheraga, H.A. (1980). "Empirical studies of hydrophobicity. 1. Effect of protein size on the hydrophobic behavior of amino acids." Macromolecules **13**: 1398-1405.

Miyazawa, S. and R. L. Jernigan (1999). "Self-consistent estimation of inter-residue protein contact energies based on an equilibrium mixture approximation of residues." Proteins **34**(1): 49-68.

Munoz, V. and L. Serrano (1994). "Intrinsic secondary structure propensities of the amino acids, using statistical phi-psi matrices: comparison with experimental scales." Proteins **20**(4): 301-11.

Naderi-Manesh, H., M. Sadeghi, et al. (2001). "Prediction of protein surface accessibility with information theory." Proteins **42**(4): 452-9.

Nishikawa, K. and T. Ooi (1986). "Radial locations of amino acid residues in a globular protein: correlation with the sequence." J Biochem **100**(4): 1043-7.

Nozaki, Y. and C. Tanford (1971). "The solubility of amino acids and two glycine peptides in aqueous ethanol and dioxane solutions. Establishment of a hydrophobicity scale." J Biol Chem **246**(7): 2211-7.

Oobatake, M. and T. Ooi (1977). "An analysis of non-bonded energy of proteins." J Theor Biol **67**(3): 567-84.

Parker, J. M., D. Guo, et al. (1986). "New hydrophilicity scale derived from high-performance liquid chromatography peptide retention data: correlation of predicted surface residues with antigenicity and X-ray-derived accessible sites." Biochemistry **25**(19): 5425-32.

Parthasarathy, S. and M. R. Murthy (2000). "Protein thermal stability: insights from atomic displacement parameters (B values)." Protein Eng **13**(1): 9-13.

Ponnuswamy, P. K. (1993). "Hydrophobic characteristics of folded proteins." Prog Biophys Mol Biol **59**(1): 57-103.

Ponnuswamy, P. K., M. Prabhakaran, et al. (1980). "Hydrophobic packing and spatial arrangement of amino acid residues in globular proteins." Biochim Biophys Acta **623**(2): 301-16.

Ptitsyn, O. B. and A. V. Finkelstein (1983). "Theory of protein secondary structure and algorithm of its prediction." Biopolymers **22**(1): 15-25.

Punta, M. and A. Maritan (2003). "A knowledge-based scale for amino acid membrane propensity." Proteins **50**(1): 114-21.

Rackovsky, S. and H. A. Scheraga (1977). "Hydrophobicity, hydrophilicity, and the radial and orientational distributions of residues in native proteins." Proc Natl Acad Sci U S A **74**(12): 5248-51.

Venanzi, T. J. (1984). "Hydrophobicity parameters and the bitter taste of L-amino acids." J Theor Biol **111**(3): 447-50.

Vihinen, M., E. Torkkila, et al. (1994). "Accuracy of protein flexibility predictions." Proteins **19**(2): 141-9.

Wold, S., Eriksson, L., Hellberg, S., Jonsson, J., Sjostrom, M., Skagerberg, B. and Wikstrom, C. (1987). "Principal property values for six non-natural amino acids and their application to a structure-activity relationship for oxytocin peptide analogues." Can. J. Chem. **65**: 1814-1820.

Zhou, H. and Y. Zhou (2004). "Quantifying the effect of burial of amino acid residues on protein stability." Proteins **54**(2): 315-22.
