# Supplementary material for: Large-scale prediction of long disordered regions in proteins using random forests
Source: BMC Bioinformatics. 2009 Jan 7;10:8. doi: 10.1186/1471-2105-10-8 (PMC2637845; doi:10.1186/1471-2105-10-8)
Supplement: Additional file 3 — Influence of the number of trees and time efficiency. Result and discussion on the influence of the number of trees and time efficiency. [file 1471-2105-10-8-S3.pdf]

Influence of the number of trees and time efficiency

It has been reported that with a larger number of trees in a random forest, rather than overfitting [2, 3] the prediction error for the forest converges to a limit [1]. The number of trees for which a forest converges depends on applications. 10-fold cross validation tests on the training data (see the Training and test datasets section) were conducted to examine the performance of IUPforest under different number of trees. The results indicated that prediction accuracy can be consistently improved with an increase in the number of trees up to 50 without significantly increasing the training time. Prediction accuracy is only modestly improved with the number of trees growing from 50 to 100, while the time for training forests significantly increased by almost 2 folds (shown in Table A2). The prediction accuracy of IUPforest under different number of trees is shown in Fig. A1. Given that 10 models need to be trained in 10-fold cross validation for tuning parameters and considering time efficiency, 50 trees are the default setting for IUPforest.

Table A2. Time for training IUPforest models under different settings of number of trees on DisProt3.6 and PDBselect25 on a machine of three i686 processors and 4GB memory running GNU Linux.
.

# Trees	Training time
10	28 mins 32.88 secs
50	120 mins 6.63 secs
100	186 mins 10.00 secs
200	360 mins 40.13 secs
300	system thrashing after 24 hours

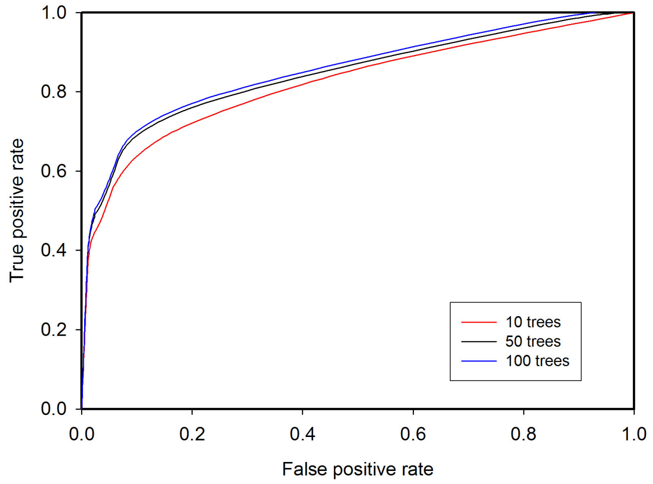

Fig. A1. The prediction accuracy of IUPforest under different number of trees in10 fold cross validation test *d =1, 2, …,15* of type I features.

References:

1. Breiman, L., *Random Forest.* Machine Learning, 2001. **45**(1): p. 5-32.
2. Han, P., et al., *Predicting disordered regions in proteins based on decision trees of reduced amino acid composition.* J Comput Biol, 2006. **13**(10): p. 1723-34.
3. Han, P., et al., *Reducing overfitting in predicting intrinsically unstructured proteins.* In Proc. The 11th Pacific-Asia Conference on Knowledge Discovery and Data Mining (PAKDD'2007), Nanjing, China, May 2007., 2007. **LNAI 4426**: p. 515-522.
