# Supplementary material for: Large-scale prediction of long disordered regions in proteins using random forests
Source: BMC Bioinformatics. 2009 Jan 7;10:8. doi: 10.1186/1471-2105-10-8 (PMC2637845; doi:10.1186/1471-2105-10-8)
Supplement: Additional file 4 — Influence of the windows and sliding step for training IUPforest-L. Results and discussions on the influence of the windows and sliding step for training IUPforest-L. [file 1471-2105-10-8-S4.pdf]

## The windows for training IUPforest

Training with small windows increases training time and can introduce noises, whereas training with large windows can loose local information. Fig. A2 shows the ROC curves for 10-fold cross validation tests on the training set with windows of 31 aa and 41 aa, and blind tests on Han-ADS1 with the IUPforest models trained with different window size. It can be seen that although independent test result on Han-ADS1 is stable between windows of 19 aa to 47 aa, 10-fold cross validation test accuracy dropped with larger window size. As a result 31 aa was set as the default window size for large-scale prediction to keep the balance between high efficiency and accuracy.

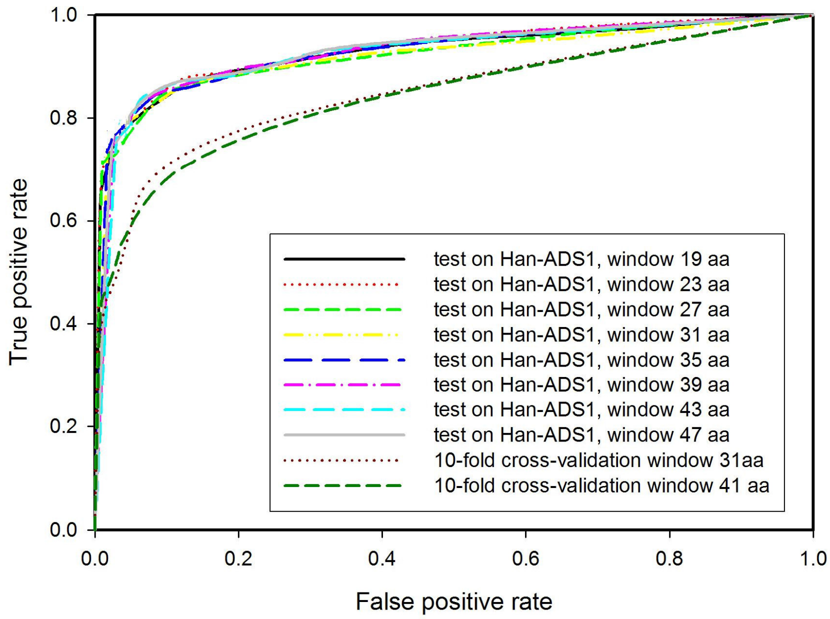


Fig A2. ROCs under different window sizes. The highest sensitivity is reached at the 10% false positive rates with windows of 19 to 47 aa on Han-ADS1 test set. On the other hand AUC will drop when window size increases from 31aa to 41 aa.

## The sliding step for training IUPforest

The step size for sliding windows can also affect the accuracy and overall time efficiency at both the training and test stage. If the step size is too small, when a window slides along a sequence, it will introduce redundancy between windows and prolong the time for training models. Fig A3 shows a 10-fold cross validation test result with sliding steps of 1aa and 20 aa

for an IUPforest model defined by type I features of only 5 sets of AAI. When a sliding step increases from 1 aa to 20 aa, the AUC will drop about 3%, but the time efficiency will increase by four folds. To ensure an efficient large-scale application, the sliding step of 20 aa was used.

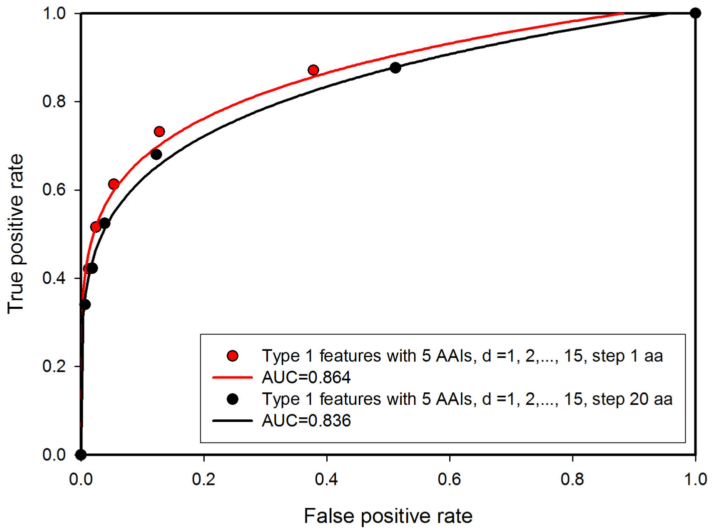


Fig A3. ROC curves of 10-fold cross validation test with 5 sets of AAI under steps of window 1 aa and 20 aa.
